# Surveillance Study of *Klebsiella pneumoniae* in the Giant Panda Revealed High Genetic Diversity and Antibiotic Therapy Challenge

**DOI:** 10.3390/antibiotics11040473

**Published:** 2022-04-01

**Authors:** Yang Feng, Yaoyan Chen, Songrui Liu, Rong Hou, Xia Yan, Yi Geng, Zhijun Zhong, Hongrui Guo, Ping Ouyang, Dongsheng Zhang, Xiaoyan Su

**Affiliations:** 1 College of Veterinary Medicine, Sichuan Agricultural University, Chengdu 611130, China; fengyang_sicau@163.com (Y.F.); scp682682@126.com (Y.C.); zhongzhijun488@126.com (Z.Z.); guohongrui@sicau.edu.cn (H.G.); ouyang.ping@live.cn (P.O.); 2 Sichuan Key Laboratory of Conservation Biology for Endangered Wildlife, Chengdu Research Base of Giant Panda Breeding, Sichuan Academy of Giant Panda, Chengdu 610081, China; srui_liu@163.com (S.L.); hourong2000@panda.org.cn (R.H.); manmananan520@126.com (X.Y.); dongsheng930206@163.com (D.Z.)

**Keywords:** *Klebsiella pneumoniae*, giant panda (*Ailuropoda melanoleuca*), surveillance study, genotype, antibiotic resistance pattern

## Abstract

*Klebsiella pneumoniae* is not only a worldwide human pathogen, it also effects wildlife, such as the giant panda (*Ailuropoda melanoleuca*), in which it has recently been evidenced to result in diarrhea, organ failure, and even death. A *K. pneumoniae* investigation was carried out at the Chengdu Research Base of Giant Panda Breeding in 2018. As part of the investigation, the pulsed-field gel electrophoresis (PFGE) typing, multilocus-sequence typing (MLST), antibiotic resistance profiles (ARPs), and antibiotic resistance genes (ARGs) were studied based on all isolates. Fecal samples were collected from 72 *A. melanoleuca* from May to December 2018, and a total of 90 *K. pneumoniae* were isolated from 153 fecal samples. The genotyping results showed that the isolates had high diversity, of which 84 clusters were obtained by PFGE and 57 STs by MLST. The overall trend of the similarity of isolates was the first sample period > second sample period > third sample period, which showed the increasement of genome variability of *K. pneumoniae*. In addition, 90 isolates showed high resistance to ampicillin, rifampicin, and compound sulfamethoxazole. Of the obtained isolates, 50% carried 6~8 ARPs, and the carrying volume increased during three sample periods, in which we found two isolates carrying 12 and 13 ARPs during the third sample period, respectively. Moreover, a total of 65 ARGs were detected (90.28%, 65/72) in 90 *K*. *pneumoniae* samples. Almost all bacteria sampled contained 17 ARGs that belonged to the β-lactamase, Multidrug, MGEs, Aminoglycoside, and Tetracycline, which may be the basis of ARPs of *K. pneumoniae*. Moreover, the types of Multidrug and MGEs had a greater impact on antibiotic susceptivity of *K. pneumoniae.* Our results showed that *K. pneumoniae* has a serious risk of transmission in *A. melanoleuca* and *K. pneumoniae* had a high possibility of genome diversity and the risk of drugs tolerance under the large antibiotic usage.

## 1. Introduction

The giant panda (*Ailuropoda melanoleuca*) is a species of *Ursidae* and is regarded as a living fossil and a national treasure of China. Although the wild population of *A. melanoleuca* has recovered to some extent and is no longer considered endangered, the species is still listed as vulnerable with the issue of disease being a serious concern for its continued survival. [1]. Diseases affecting *A. melanoleuca* can be caused by parasites (e.g., ascariasis and fluke disease) [2], bacteria (e.g., *Klebsiella pneumoniae*, *Escherichia coli*, and *Pseudomonas aeruginosa*) [3,4,5,6], and viruses (e.g., canine distemper and rotavirus) [7,8]. *K. pneumoniae* has recently been revealed to be an important opportunistic pathogen that infects giant pandas [4,9,10]. The pathogenic effects of *K. pneumoniae* on *A. melanoleuca* were clinically divided into two types: the enteritis type, which caused lethargy, loss of appetite, gradual emaciation, diarrhea, mucosal feces, and exfoliated and inflamed intestinal mucosa [11]; and the septic type, which caused the systemic infection, including the degeneration of multiple organs, necrosis, sepsis, and even death [12]. Thus, preventing *K. pneumoniae* infection is an important aspect of the conservation of this vulnerable species.

In recent years, antibiotic-resistant bacteria and antibiotic resistance genes (ARGs) have been labeled as environmental contaminants. According to the WHO’s “One Health” theory, animals and the environment are important carriers of antibiotic resistance, and there is an increase in zoonotic diseases, sometimes leading to deadly epidemics. Studies have shown that ARGs can be transmitted between humans and animals and can even remain in the environment [13,14]. More importantly, bacteria have been shown to readily share ARGs by horizontal gene transfer via mobile genetic elements, including plasmids, transposons, and integrons [15,16]. The rapid increase in the prevalence of multidrug-resistant *K. pneumoniae* presents a challenge to clinical treatment and public health [17]. However, the characteristics of antibiotic resistance of *K. pneumoniae* found in *A. melanoleuca* are not clear at present. As a result, it is unknown which antibiotics can be used safely or whether they could be used continually in this species.

**Figure 1:**
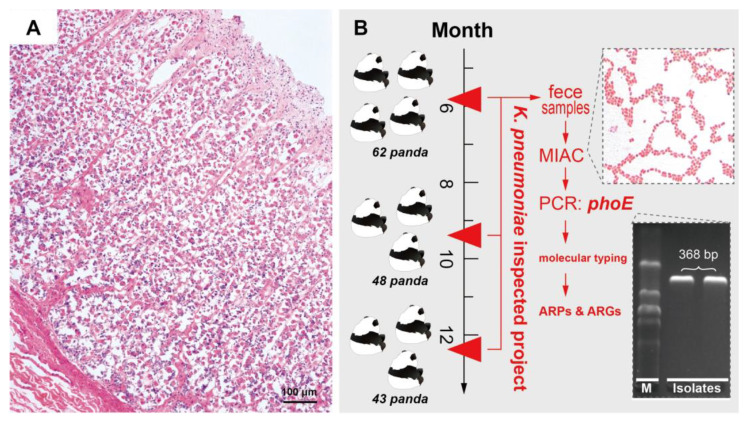
Characteristics of collected samples. (**A**) Histopathology showing the degeneration and necrosis of intestinal mucosa (sample was obtained from a dead *A. melanoleuca* and diagnosed as *K. pneumoniae*, while the case and histologic technical was referring to a previous study [12]). (**B**) The scheme of the *K. pneumoniae* inspected project.

The Chengdu Research Base of Giant Panda Breeding (CRBGPB) is a world-famous ex-situ conservation base for *A. melanoleuca*. By the end of 2020, there were 215 *A. melanoleuca* at the CRBGPB. To prevent further spread of the *K. pneumoniae* infection (Figure 1B), and to prevent the possibility of zoonotic infection, an investigation and treatment program was initiated. The aim of this investigation was to understand the molecular epidemiological characteristics of *K. pneumoniae*, such as the distribution in *A. melanoleuca*, and the origin and drug resistance characteristics, through pulsed-field gel electrophoresis (PFGE) typing, multilocus sequence typing (MLST), antibiotic resistance profiles (ARPs), and ARGs.

## 2. Results

### 2.1. Isolation of K. pneumoniae

Ninety strains of *K. pneumoniae* were isolated from 153 fecal samples through the three different sample periods (a total of 72 *A. melanoleuca* and partial *A. melanoleuca* were replicated, sampled in different periods) (Table 1). The results showed that *K. pneumoniae* has a high distribution in *A. melanoleuca*, and the isolation rate was 59.68% (37/62) for the first sample period, 83.33% (40/48) for the second sample period, and 30.23% (13/43) for the third sample period. 

### 2.2. Genotype Characteristics of K. pneumoniae by PFGE and MLST

**Figure 2:**
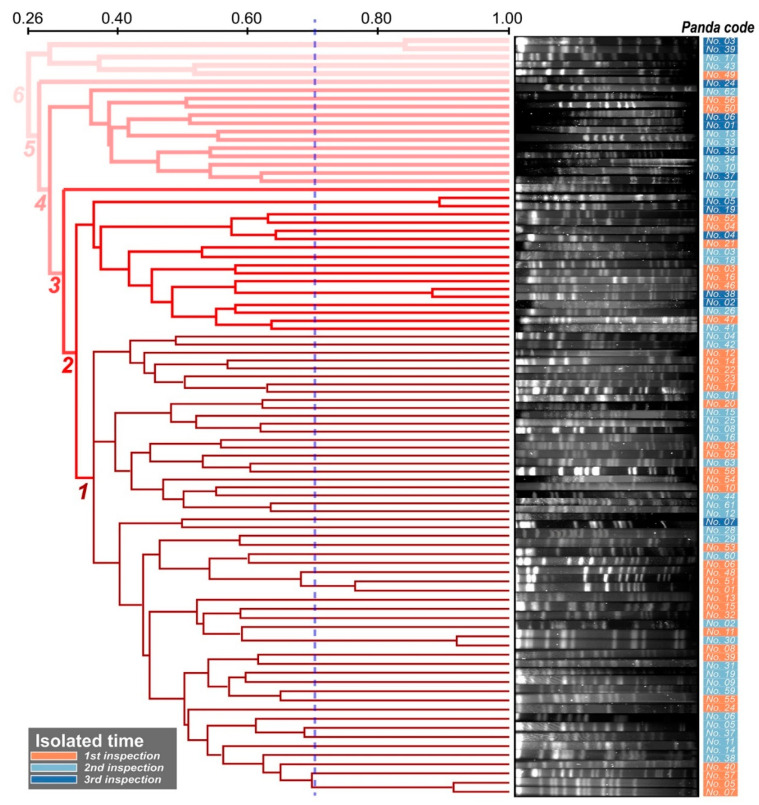
Dendrogram of the PFGE data of the 90 *K. pneumoniae* strains. Similarities of ≥75.0% were considered as the same cluster. The thickness of the lines makes it easy to comprehend the distance of the dendrogram.

**Figure 3:**
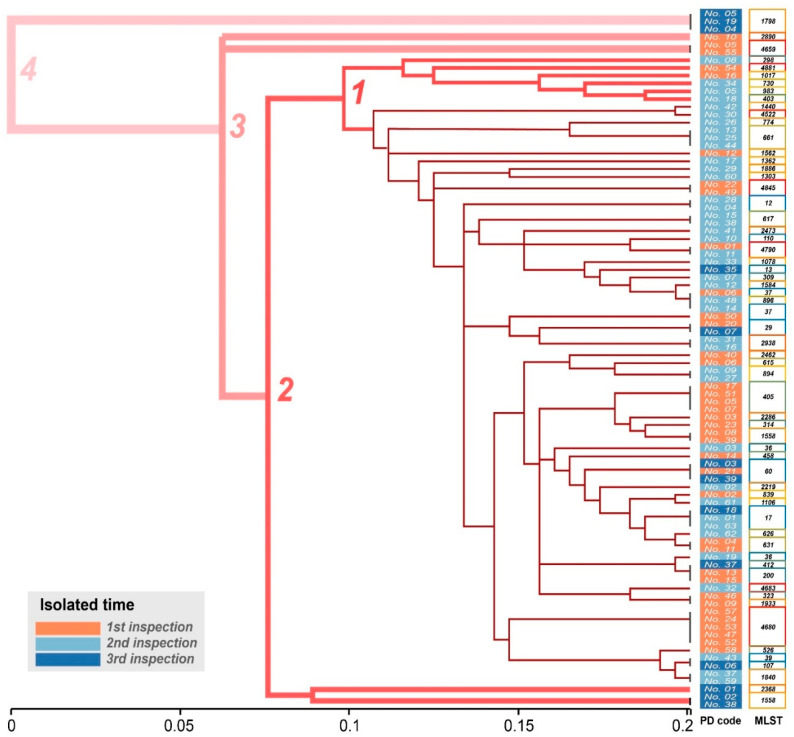
Hierarchical clustering analysis of 90 *K. pneumoniae* strains. The thickness of the lines makes it easy to comprehend the distance of the dendrogram.

The PFGE results showed that *K. pneumoniae* strains could be divided into 84 clusters, which showed a high diversity of the genome from all isolates (Figure 2). Additionally, *K. pneumoniae* isolated from the same individual at different times showed little similarity, indicating that *A. melanoleuca* are at risk of reinfection with *K. pneumoniae* or the effortless mutation of strains under therapeutic pressure. However, we found a more interesting point: the bacteria isolated from the first and second sample period had a higher similarity compared with the third sample period (Figure 2). The branch of the dendrogram was divided into six stages. In total, 91.9% of the strains (34/37) lay in the 1~2 branch stages in the first sample period, while 80% of the strains (32/40) were in these stages in the second sample period, which was reduced to 46.2% (6/13) during the third sample period. Moreover, the MLST analysis yielded similar results to the PFGE analysis (Figure 3, Table S1). A total of 57 sequence types (STs) were obtained from the 90 isolates through the MLST analysis, and the proportions of strains in the three inspections in the first branch (divided into four stages) were 91.9% (34/37), 100% (40/40), and 53.8% (7/13), which also proved the high diversity of the genome of all isolates and was similar to the PFGE results (Figure 3, Table S1).

### 2.3. The Antibiotic Resistance Profiles of K. pneumoniae

**Figure 4:**
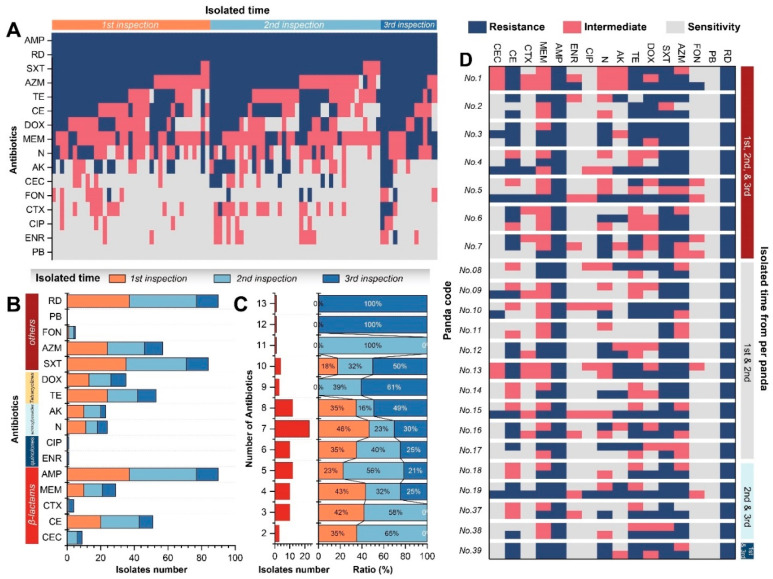
The ARPs of *K. pneumoniae* strains isolated from *A. melanoleuca*. (**A**) Distribution of resistance in all *K. pneumoniae* strains. (**B**) Statistics of the classification of 15 antibiotics. (**C**) Percentage accumulation of different antibiotic resistances during the different sample periods. (**D**) Comparison of the ARPs per individual. Abbreviations: cefaclor (CEC), cefotaxime (CTX), cephradine (CE), meropenem (MEM), ampicillin (AMP), ciprofloxacin (CIP), enrofloxacin (ENR), neomycin (N), amikacin (AK), cotri-moxazole (SXT), doxycycline (DOX), tetracycline (TE), azithromycin (AZM), florfenicol (FON), rifampicin (RD), and polymyxin B (PB).

The ARP showed that 90 isolates had high resistance to ampicillin (AMP), rifampicin (RD), and cotrimoxazole (SXT), but low resistance to cefaclor (CEC), florfenicol (FON), cefotaxime (CTX), ciprofloxacin (CIP), enrofloxacin (ENR), and polymyxin B (PB) and moderate resistance to the other six antibiotics (Figure 4A). Additionally, the overall resistance had a similarity from the three sample periods; however, the strains isolated from the second and third sample periods appeared to be resistant to CEC and CTX, while strains isolated from the third sample period appeared to be resistant to CIP and ENR (Figure 4A). According to the classification of 15 antibiotics, *K. pneumoniae* isolated from the CRBGPB may have a high potential resistance to β-lactams, tetracycline, macrolides, and sulfonamides (Figure 4B). Moreover, the multi-antibiotics resistance analysis showed that 90 isolates carried 2 to 13 ARPs, with the largest number of bacteria with 7 ARPs (23 strains), which was consistent with the normal distribution (Figure 4C). Furthermore, the maximum number of ARPs in the *K. pneumoniae* isolated from the first sample period was 10, the isolates in the second sample period increased to 11 ARPs, and the isolates in the third sample period increased to 13 ARPs. The results showed that the antibiotic resistance of *K. pneumoniae* in *A. melanoleuca* showed an increase over time (Figure 4C). Finally, we compared the changes of ARPs of *K. pneumoniae* isolated from the same individual at different times, which showed great differences in response to doxycycline (DOX), azithromycin (AZM), neomycin (N), cephradine (CE), meropenem (MEM), and tetracycline (TE), which belong to different antibiotic classifications (Figure 4D). 

### 2.4. The Antibiotic Resistance Genes of K. pneumoniae

**Figure 5:**
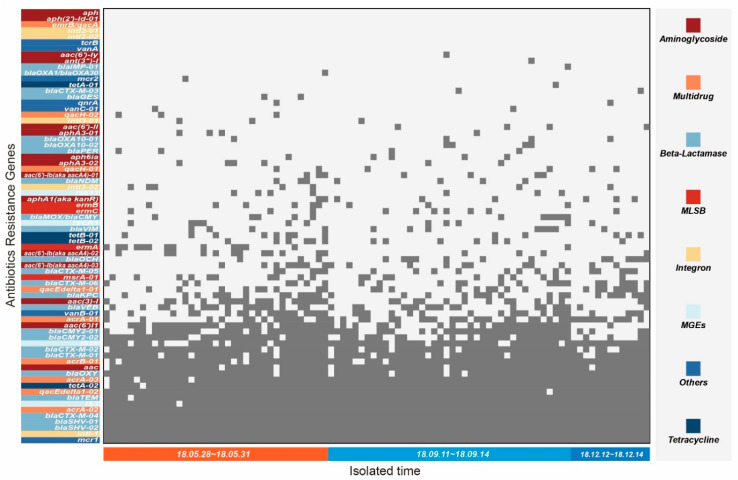
Positive detection of ARGs in *K. pneumoniae* strains isolated from *A. melanoleuca*. The gray dots represent the corresponding ARGs detected in the strain.

**Figure 6:**
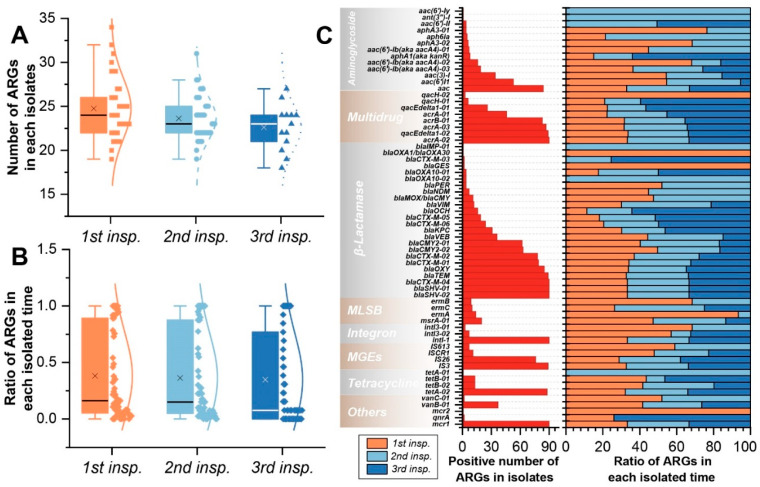
The ARGs statistics of *K. pneumoniae* strains isolated from *A. melanoleuca*. (**A**) Number of ARGs in each isolated time. (**B**) Ratio of ARGs in each isolated time. (**C**) Distribution and percentage accumulation of ARGs in each isolated time.

A total of 65 ARGs were detected (90.28%, 65/72) in 90 *K*. *pneumoniae* strains. Almost all the bacteria sampled contained 17 ARGs, including *mcr1*, *intl-1*, *blaSHV-02*, *blaSHV-01*, *blaCTX-M-04*, *acrA-02*, *IS3*, *blaTEM*, *qacEdelta1-02*, *tetA-02*, *acrA-03*, *blaOXY*, *aac*, *acrB-01*, *blaCTX-M-01*, *blaCTX-M-02*, and *IS26*, with a detection rate between 84.44%~100% (Figure 5). These ARGs belonged to the antibiotic resistance mechanism, including β-lactamase, Multidrug, Mobile genetic elements (MGEs), Aminoglycoside, and Tetracycline (Figure 5), which may be the basis of ARPs of 90 strains of *K. pneumoniae*, while the ARGs in types of MGEs, tetracycline, and MLSB were lower than these five types. Notably, 41.1% *K. pneumoniae* strains also carried a ARG *vanB-01*, which is resistant to vancomycin, an antibiotic commonly used to treat Gram-positive bacteria. In addition, different bacteria carried a range of 18~34 ARGs (median: 24), but there was no significant difference among different sample periods (Figure 6A). There was no significant difference in the carrying rate of all ARGs at different sample periods either (Figure 6B). Moreover, the carrying proportion was similar in the three inspections for some ARGs with high distribution rates in strains, but ARGs with low distribution were disordered in the three sample periods (Figure 6C).

### 2.5. Correlation Analysis Based on Detected Parameters of Strains

**Figure 7:**
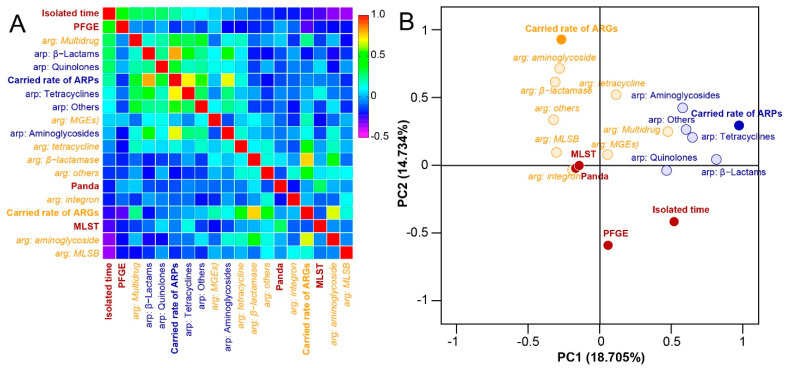
Correlation analysis based on detected parameters of strain. (**A**) Results of cluster analysis on the indexes of detected parameters. (**B**) Principal components analysis (PCA) analysis of detected parameters. The principal component regression analysis (fixed dimension: 2) of the correlation matrix was used to calculate the characteristic value of each detected parameters using SPSS 20.0 (IBM, New York, NY, USA).

In this study, the relationship between the detected parameters (PFGE, MLST, ARPs, and ARGs) of strains was further discussed (Figure 7A,B). Bivariate correlation analysis showed that PFGE had a high correlation with the isolated time of strains (correlation coefficient = 0.52). There was a significant correlation among different types of ARGs or different types of ARPs, and the correlation between different ARPs was also related to ARG type Multidrug (CC = 0.218 ± 0.084) and MGEs (CC = 0.094 ± 0.031). The results further showed that *K. pneumoniae* had a high genetic structure variation, and this structure (MLST) was associated with ARGs, while ARG type Multidrug and MGEs had a greater impact on antibiotic susceptivity of *K. pneumoniae* (Figure 7A,B). 

## 3. Discussion

*K. pneumoniae* is an important zoonotic bacterial pathogen worldwide. It causes nosocomial infections, as well as infections in animals [4,18,19]. Antibiotics are the main choice for the treatment and prevention of bacterial disease, but because of misuse, bacterial resistance has increased [20]. More insight is needed to improve our understanding of this pathogen, the analysis of its resistance genes, and its genetic diversity. A *K. pneumoniae* investigation was carried out during 2018. A total of 72 *A. melanoleuca* participated in this investigation, and a total of 90 *K. pneumoniae* strains were isolated from 153 fecal samples during three sample periods. The genotyping results showed that the isolates had high diversity, of which 84 clusters were obtained by PFGE and 57 STs by MLST. The overall trend of the similarity of isolates was first sample > second sample > third sample., which showed the increase of genome variability of *K. pneumoniae*. In addition, these 90 isolates showed high resistance to ampicillin, rifampicin, and compound sulfamethoxazole. Of the obtained isolates, 50% carried 6~8 ARPs, and the carrying volume was increased during the three sample periods. We also found that two isolates carried 12 and 13 ARPs during the third sample period, respectively, which had little similarity with the ARGs of *Shigella* isolated from *A. melanoleuca* [21]. Moreover, a total of 65 ARGs were detected (90.28%, 65/72) in 90 *K*. *pneumoniae* strains. Almost all bacteria contain 17 ARGs that belong to the β-lactamase, Multidrug, MGEs, Aminoglycoside, and Tetracycline, which may be the basis of ARPs of *K. pneumoniae.* Our project implementation revealed that *K. pneumoniae* has a great risk of transmission in *A. melanoleuca*. 

The *blaCTX-M-4*, *blaSHV-1*, *blaSHV-2*, *intI-1*, *acrA-2*, *mcr1*, *blaTEM*, *IS3*, *qacEderta1-2*, and *tetA-2* genes were prevalent in the *A. melanoleuca* samples. Frequent epidemics of the *blaCTX-M* gene have been reported in France and Italy [22,23], *blaSHV-01* and *blaTEM* genes are major epidemic genes in the United States and Iran [24], and the *blaKPC* gene was widely prevalent in a hospital in China [25]. The regional characteristics of different ARGs indicate the source of multiple strains of *K. pneumoniae* deserve evaluation [26]. Additionally, the genotypes of the 90 strains of *K. pneumoniae* isolated from *A. melanoleuca* showed high diversity. *K. pneumoniae* infection is a zoonotic disease, and it can be carried or transmitted by tourists and keepers working with *A. melanoleuca* around the world. Thus, we hypothesized that they may be caused by exogenous input and cross-infection. The CRBGPB receives up to 9 million domestic and international visitors a year. We feel that the reasons for this high degree of genetic variation deserve further investigation to determine whether there is a risk of exogenous access at the CRBGPB and to detect the carrier rate and resistance characteristic of *K. pneumoniae* among caregivers and tourists [27].

*K. pneumoniae* plays a key role in disseminating ARGs from environmental microbes to clinically-important pathogens [28]. The “One Health” theory claims that animals and the environment are an important part of the development of antimicrobial resistance. For the sake of public health, humans need to pay attention to diseases and drug resistance from animals to control or prevent disease outbreaks through integrated public health, veterinary, animal management, and an ecological approach [29]. *K. pneumoniae* is likely to be found associated with human, animal, and environmental vectors; therefore, it is necessary to monitor the resistance of *K. pneumoniae* from the *A. melanoleuca* and regulate the use of antibiotics to avoid epidemics in clinical practice. According to the results of this study, *K. pneumoniae* generally possesses ARGs with multiple antibiotic resistance mechanisms, which proves the theoretical possibility of multiple antibiotic resistance. Additionally, we found that, in the first sample period, there was one isolate carrying 10 ARPs. In the second sample period, there appeared to be not only two isolates carrying 10 ARPs but also one isolate carrying 11 ARPs. Lastly, in the third sample period, there were isolates carrying 12 and 13 ARPs. This suggests that there has been a significant increase in the multiple antibiotic resistance of *K. pneumoniae* in *A. melanoleuca*, with the potential risk of developing superbugs.

## 4. Materials and Methods

### 4.1. Bacterial Isolation and Identification

The investigation of *K. pneumoniae* was carried out in the CRBGPB, and the sample time was concentrated in three periods: first (18 May 2018~31 May 2018), second (11 September 2018~14 September 2018), and third (12 December 2018~14 December 2018) (Figure 1B). In the experiment, bacteria were isolated from fresh feces, following the methods described in Liu et al. [21] (the time from defecation to freezing fixation was <10 min). In brief, under aseptic conditions, sterile cotton swabs were used to take the central portion of the feces, scrape part of the feces, and dissolve this in aseptic water. The supernatant was taken and inoculated in Luria-Bertani (LB) broth and shaken at 37 °C for 16~18 h. After initial isolation using Mark Kang Kai inositol adonitol carbenicillin (MIAC) Agar Base, all isolates were identified using the *16S rDNA* gene (27F: 5′-AGAGTTTGATCCTGGCTCAG-3′; 1492R: 5′-GGCTACCTTGTTACGACTT-3′) and *phoE* gene (F: 5′-TGGCCCGCGCCCAGGGTTCGAAA-3′; R: 5′-GATGTCGTCATCGTTGATGCCGAG-3′) [30] sequence analysis. The identified strains were inoculated in LB broth at 37 °C for 24 h and then freeze-dried with skim milk powder and preserved at −80 °C.

### 4.2. Pulsed Field Gel Electrophoresis Analysis

PFGE was performed using the PulseNet protocol with minor modifications [31]. The isolates were grown on LB agar at 37 °C for 24–48 h. Single colonies from plates were then suspended with sterile cotton swabs in 5 mL of cell suspension buffer (100 mM Tris, 100 mM EDTA, pH 8.0). The optical density was then adjusted to 0.8–1.0 at 610 nm, and measurements were made using a 2500 model Shimadzu spectrophotometer (Shimadzu, Chiyoda-Ku, Tokyo, Japan). In each tube, 400 μL of cell suspension was gently mixed with 400 μL of 1.0% SeaKem Gold agarose (Lonza Walkersville Inc., Walkersville, MD, USA) and 40 μL of proteinase K (20 mg/mL), which was then dispensed into plug molds (Bio-Rad Laboratories). The plugs were then transferred to 50-mL sterile tubes, each containing 5 mL of cell lysis buffer (50 Mm Tris; 50 mM EDTA; 1% SDS; pH 8.0) and 25 μL of proteinase K (20 mg/mL) and were incubated for 3–4 h at 55 °C in a shaking incubator set at 150–175 rpm. The plugs were then washed four times with preheated (55 °C) DNase-free dH_2_O, followed by three washes with TE buffer (10 mM Tris; 1 mM EDTA, pH 8.0). After the final wash, the plugs were stored in TE buffer at 4 °C.

The agarose plugs were digested with the restriction endonuclease XbaI (50U) (Takara Biotechnology (Dalian) Co., Ltd., Dalian, China) for 4 h at 37 °C and were loaded into 1% Seakem Gold agarose. The electrophoresis was run in CHEF Mapper^®^ XA (Bio-Rad Laboratories. Beijing, China), with a pulse ramping between 6–36 s at 14 °C for 18.5 h and 6 V/cm. The PFGE results were analyzed using Quantity One v.4.62 (BioRad Laboratories. Beijing, China), and a dendrogram was created from a matrix of band matching using the unweighted pair group method using arithmetic averages (UPGMA). According to the guidelines for interpreting chromosomal DNA restriction patterns produced by PFGE [32], patterns with ≥75.0% similarity (fewer than three bands of difference) were considered the same cluster with closely- or possibly-related isolates.

### 4.3. Multilocus Sequence Typing Analysis

PCR amplification of seven housekeeping genes (*glyceraldehyde 3-phosphate dehydrogenase* [*gapA*], *translation initiation fact or 2* [*infB*], *malate dehydrogenase* [*mdh*], *phosphoglucose isomerase* [*pgi*], *phosphorine E* [*phoE*], *beta-subunit of RNA polymerase* [*rpoB*], and *periplasmic energy transduce* [*tonB*]) was performed on all isolates, as previously described (Table S2) [33]. For PCR, the 25 μL reaction mixture contained 12.5 μL SYBR Green PCR Master Mix, 8.5 μL diethylpyrocarbonate-treated water, 1.0 μL of forward primer, 1.0 μL of reverse primer, and 2 μL DNA. The following program conditions were used for the reactions: 2 min at 94 °C for 1 cycle, samples were amplified for 35 cycles at 94 °C for 30 s, 50 °C (all genes) for 1 min, followed by 72 °C for 30 s. The universal primers of the *K. pneumoniae* housekeeping gene (F: 5′-GTTTTCCCAGTCACGACGTTGTA-3′; R: 5′-TTGTGAGCGGATAACAATTTC-3′) were used for sequencing. The allelic profiles and sequence type (ST) of the sequence of all housekeeping genes from *K. pneumoniae* were assigned at BIGSdb-Pasteur (Institut Pasteur/*Klebsiella pneumoniae*. Available online: http://bigsdb.pasteur.fr/klebsiella/, accessed on 1 March 2022).

### 4.4. Antibiotic Resistance Profiles Assay

The antibiotic susceptibility of each isolate was determined using the disc diffusion method and the criteria specified by the Clinical and Laboratory Standards Institute (CLSI). Discs (Hangzhou Taihe Microbiological Reagent, Hangzhou, China) of 15 anti-microbial agents were used, including CEC, CTX, CE, MEM, AMP, CIP, ENR, N, AK, SXT, DOX, TE, AZM, FON, PB, and RD (Table S3). The sensitivity and resistance of each isolate were determined following the manufacturer’s instructions. According to the CLSI 2015 test standard, the determination results were divided into sensitivity (S), intermediate (I), and resistance (R) [34]. 

### 4.5. Antibiotic Resistance Gene Detection

Bacterial genomic DNA was extracted using a DNA extraction kit (TaKaRa, Dalian, China). The quality of DNA was checked by spectrophotometric analysis using NanoDrop ND-2000 (Nanodrop, Wilmington, DE, USA), and the concentration of DNA was determined using a QuantiFluor^®^ dsDNA system (Promega, Madison, WI, USA) using fluorometric analysis with a microplate reader (SpectraMax^®^ M5, Molecular Devices, San Jose, CA, USA). The DNA was stored at −20 °C until use. High-throughput qPCR reactions were performed using the Wafergen SmartChip Real-time PCR system (WaferGen, Fremont, CA, USA). A total of 72 ARGs were detected in this study (Table S4). Three reactions were run in parallel for each sample. After the initial enzyme activation at 95 °C for 10 min, 40 cycles of the following program were used for amplification: denaturation at 95 °C for 30 s and annealing at 60 °C for 30 s. The results were then analyzed with SmartChip qPCR Software to exclude the wells with multiple melting peaks or amplification efficiency beyond the range (90–110%). Thirty-one cycles of C_t_ value was set as the threshold value, with two or more of the three replicates detected while deviations < 20%, meeting the curve fitting analysis, were judged to be detected positive. In this project, all 16S rRNA genes in all samples with concentrations ≥ 10 ng/uL were detected positive, and NTC was not amplified, which proved that the experimental effect was good and the results were credible. The relative Copy Number = copy_(gene)_/copy_(16S)_ was used to calculate relative changes in gene expression from the qPCR results (Copy = 10^(31 − C_t_)/(10/3)^). When the C_t_ value was detected negatively, it was replaced by 31 and the Copy value would adjust to 1. Additionally, Log_10_(Relative Copy Number × 10^6^) was performed for subsequent analysis. Variables were presented as frequencies and were analyzed using the chi-square (χ2) test. Moreover, the ARPs and ARGs isolated strains were classified according to region, time, and subtype, presented as frequencies and analyzed using the chi-square (χ2) test.

## 5. Conclusions

This study has provided an investigation of *K. pneumoniae* carried by *A. melanoleuca* in the CRBGPB. The comprehensive genotype and antibiotic resistance characteristics revealed the high genome diversity and high antibiotic resistance of *K. pneumoniae* and its potential relevance to animal health and human public health. Ultimately, the information presented in this study will help with the development of guidance for the ex-situ conservation of *A. melanoleuca*. 

## Figures and Tables

**Table 1:** Details of bacterial isolation.

Panda Code	1st Time	2nd Time	3rd Time	Pos./Total	Panda Code	1st Time	2nd Time	3rd Time	Pos./Total
*No. 1*	+	+	+	3/3	*No. 37*	ND	+	+	2/2
*No. 2*	+	+	+		*No. 38*	ND	+	+	
*No. 3*	+	+	+		*No. 39*	+	ND	+	
*No. 4*	+	+	+		*No. 40*	+	ND	−	1/2
*No. 5*	+	+	+		*No. 41*	−	+	ND	
*No. 6*	+	+	+		*No. 42*	−	+	ND	
*No. 7*	+	+	+		*No. 43*	−	+	ND	
*No. 8*	+	+	−	2/3	*No. 44*	−	+	ND	
*No. 9*	+	+	−		*No. 45*	−	ND	−	0/2
*No. 10*	+	+	−		*No. 46*	+	ND	ND	1/1
*No. 11*	+	+	−		*No. 47*	+	ND	ND	
*No. 12*	+	+	−		*No. 48*	+	ND	ND	
*No. 13*	+	+	−		*No. 49*	+	ND	ND	
*No. 14*	+	+	−		*No. 50*	+	ND	ND	
*No. 15*	+	+	−		*No. 51*	+	ND	ND	
*No. 16*	+	+	−		*No. 52*	+	ND	ND	
*No. 17*	+	+	−		*No. 53*	+	ND	ND	
*No. 18*	−	+	+		*No. 54*	+	ND	ND	
*No. 19*	−	+	+		*No. 55*	+	ND	ND	
*No. 20*	+	−	−	1/3	*No. 56*	+	ND	ND	
*No. 21*	+	−	−		*No. 57*	+	ND	ND	
*No. 22*	+	−	−		*No. 58*	+	ND	ND	
*No. 23*	+	−	−		*No. 59*	ND	+	ND	
*No. 24*	+	−	−		*No. 60*	ND	+	ND	
*No. 25*	−	+	−		*No. 61*	ND	+	ND	
*No. 26*	−	+	−		*No. 62*	ND	+	ND	
*No. 27*	−	+	−		*No. 63*	ND	+	ND	
*No. 28*	−	+	−		*No. 64*	−	ND	ND	0/1
*No. 29*	−	+	−		*No. 65*	−	ND	ND	
*No. 30*	−	+	−		*No. 66*	−	ND	ND	
*No. 31*	−	+	−		*No. 67*	−	ND	ND	
*No. 32*	−	+	−		*No. 68*	−	ND	ND	
*No. 33*	−	+	−		*No. 69*	−	ND	ND	
*No. 34*	−	+	−		*No. 70*	ND	−	ND	
*No. 35*	−	−	+		*No. 71*	ND	ND	−	
*No. 36*	−	−	−	0/3	*No. 72*	ND	ND	−	
right continued					pos./total	37/62	40/48	13/43	

+: *K. pneumoniae* detected positive; −: *K. pneumoniae* detected negative; N(D): no detection.

## Data Availability

The ST of the sequence from *K. pneumoniae* were assigned at Institut Pasteur/*Klebsiella pneumoniae*. Available online: http://bigsdb.pasteur.fr/klebsiella/, accessed on 1 March 2022.

## Supplementary Materials

The following supporting information can be downloaded at: https://www.mdpi.com/article/10.3390/antibiotics11040473/s1, Table S1: Allele number and MLST types of 90 *K. pneumoniae*; Table S2: Primer sequence information of housekeeping gene of *K. pneumoniae*; Table S3: Determination criteria for antibiotic resistance profiles; Table S4: Primers information of antibiotic resistance genes (ARGs).
