# Supplementary material for: Surveillance Study of Klebsiella pneumoniae in the Giant Panda Revealed High Genetic Diversity and Antibiotic Therapy Challenge
Source: Antibiotics (Basel). 2022 Apr 1;11(4):473. doi: 10.3390/antibiotics11040473 (PMC9030629; doi:10.3390/antibiotics11040473)
Supplement: Supplementary file 1 [file antibiotics-11-00473-s001.zip › antibiotics-1636381-supplementary.pdf]

Table S1 Allele number and MLST types of 90 *K. pneumoniae*

Panda code	Isolated time	*gapA*	*infB*	*mdh*	*pgi*	*phoE*	*rpoB*	*tonB*	MLST
No. 09	18.05.28~18.05.31	2	1	1	1	3	1	9	1933
No. 03		2	1	62	1	10	4	110	2286
No. 47		2	1	1	2	2	1	43	4680
No. 46		2	1	1	1	9	1	93	323
No. 21		2	1	2	1	4	4	8	60
No. 50		2	9	2	1	13	45	16	37
No. 05		2	1	62	3	10	4	110	405
No. 04		2	1	1	1	9	4	4	631
No. 52		2	1	1	2	2	1	43	4680
No. 07		2	1	62	3	10	4	110	405
No. 16		2	2	2	1	1	4	34	1017
No. 56		4	114	1	1	13	70	12	4659
No. 10		2	115	114	166	2	146	140	2890
No. 20		2	3	2	2	6	4	4	29
No. 01		2	3	1	8	12	1	22	4790
No. 22		2	1	62	3	10	4	4	4845
No. 02		3	1	1	1	9	4	177	839
No. 48		2	9	2	1	13	1	38	896
No. 14		2	1	2	2	10	4	9	458
No. 13		2	1	2	1	12	1	68	200
No. 51		2	1	62	3	10	4	110	405
No. 15		2	1	2	1	12	1	68	200
No. 23		2	1	5	2	7	4	23	314
No. 40		16	18	43	49	47	30	75	2462
No. 53		2	1	1	2	2	1	43	4680
No. 24		2	1	1	2	2	1	43	4680
No. 58		38	19	53	58	73	21	130	526
No. 54		43	3	1	1	1	4	91	4881
No. 55		4	114	1	1	13	70	12	4659
No. 57		2	1	1	2	2	1	43	4680
No. 08		4	1	6	1	12	5	30	1558
No. 11		2	1	1	1	9	4	4	631
No. 12		16	24	36	27	29	22	105	1562
No. 49		2	1	62	3	10	4	4	4845
No. 39		4	1	6	1	12	5	30	1558
No. 17		2	1	62	3	10	4	110	405
No. 06		16	1	21	76	47	22	152	615
No. 06	18.9.11~18.9.14	2	9	2	1	13	1	16	37
No. 14		2	9	2	1	13	1	16	37
No. 19		2	1	2	1	7	1	7	36
No. 43		2	1	2	4	9	1	16	39
No. 15		2	9	1	1	13	1	2	617
No. 30		120	9	2	1	13	1	16	4522
No. 05		4	1	2	1	1	4	62	983
No. 38		2	9	1	1	13	1	2	617
No. 42		10	3	2	1	4	1	4	1440
No. 17		2	1	65	1	10	11	2	1362
No. 02		2	1	1	2	16	4	337	2219
No. 12		18	22	18	16	25	13	165	1584
No. 25		4	3	1	36	9	10	14	661
No. 27		18	15	18	61	93	37	99	894
No. 59		18	15	18	22	31	13	51	1840
No. 60		2	5	118	1	179	1	13	1303
No. 61		2	1	1	1	7	2	12	1106
No. 01		2	1	1	1	4	4	4	17
No. 62		3	1	1	1	27	5	25	626
No. 29		2	5	118	1	10	1	13	1886
No. 09		18	15	18	61	93	37	99	894
No. 11		2	3	1	8	12	1	22	4790
No. 31		2	3	2	1	7	4	12	2938
No. 32		2	1	1	1	48	1	46	4683
No. 37		18	15	18	22	31	13	51	1840
No. 33		4	5	1	3	12	4	46	1078
No. 18		2	1	2	1	1	7	13	403
No. 03		2	1	2	1	7	45	7	36

No. 26		2	1	1	17	4	16	4	774
No. 07		2	9	2	1	13	1	10	309
No. 16		2	3	2	1	7	4	12	2938
No. 44		4	3	1	36	9	10	14	661
No. 04		6	3	1	1	12	45	4	12
No. 28		6	3	1	1	12	45	4	12
No. 08		2	1	2	1	1	1	7	298
No. 41		2	51	1	4	17	15	355	2473
No. 10		2	6	1	3	8	1	44	110
No. 13		4	3	1	36	9	10	14	661
No. 63		2	1	1	1	4	4	4	17
No. 34		2	1	33	1	1	4	56	730
No. 35	18.12.12~18.12.14	2	3	1	1	56	1	110	13
No. 39		2	1	2	1	4	4	8	60
No. 03		2	1	2	1	4	4	8	60
No. 37		2	1	2	1	9	1	112	412
No. 18		2	1	1	1	4	4	4	17
No. 01		16	115	370	166	47	146	606	2368
No. 07		2	3	2	2	6	4	4	29
No. 19		3	5	1	134	10	4	4	1798
No. 05		3	5	1	134	10	4	4	1798
No. 04		3	5	1	134	10	4	4	1798
No. 06		2	1	2	17	27	1	39	107
No. 02		2	1	4	14	1	5	30	1558
No. 38		2	1	4	14	1	5	30	1558

Table S2 Primer sequence information of housekeeping gene of *K. pneumoniae*

Housekeeping Gene	abbreviate	Primer Sequence	Length (bp)
beta-subunit of RNA polymerase	*rpoB*	F: Vic3oF: GTTTTCCCAGTCACGACGTTGTA**GGCGAAATGGCWGAGAACCA**	501
		R: Vic2oR: TTGTGAGCGGATAACAATTTC**GAGTCTTCGAAGTTGTAACC**	
glyceraldehyde 3-phosphate dehydrogenase	*gapA*	F: gapA173oF: GTTTTCCCAGTCACGACGTTGTA**TGAAATATGACTCCACTCACGG**	450
		R: gapA181oR: TTGTGAGCGGATAACAATTTC**CTTCAGAAGCGGCTTTGATGGCTT**	
malate dehydrogenase	*mdh*	F: mdh130oF: GTTTTCCCAGTCACGACGTTGTA**CCCAACTCGCTTCAGGTTCAG**	477
		R: mdh867oR: TTGTGAGCGGATAACAATTTC**CCGTTTTTCCCCAGCAGCAG**	
phosphoglucose isomerase	*pgi*	F: pgi1FoF: GTTTTCCCAGTCACGACGTTGTA**GAGAAAAACCTGCCTGTACTGCTGGC**	432
		R: pgi1RoR: TTGTGAGCGGATAACAATTTC**CGCGCCACGCTTTATAGCGGTTAAT**	
phosphorine E	*phoE*	F: phoE604.1oF: GTTTTCCCAGTCACGACGTTGTA**ACCTACCGCAACACCGACTTCTTCGG**	420
		R: phoE604.2oR: TTGTGAGCGGATAACAATTTC**TGATCAGAACTGGTAGGTGAT**	
translation initiation fact or 2	*infB*	F: infB1FoF: GTTTTCCCAGTCACGACGTTGTA**CTCGCTGCTGGACTATATTCG**	318
		R: infB1RoR: TTGTGAGCGGATAACAATTTC**CGCTTTCAGCTCAAGAACTTC**	
periplasmic energy transducer	*tonB*	F: tonB1FoF: GTTTTCCCAGTCACGACGTTGTA**CTTTATACCTCGGTACATCAGGTT**	414
		R: tonB2RoR: TTGTGAGCGGATAACAATTTC**ATTCGCCGGCTGRGCRGAGAG**	

Table S3 Determination criteria for antibiotic resistance profiles

Types	Antibiotics	Content (μg/tablet)	Diameter (mm)		
			Resistance (R)	Intermediate (I)	Sensitivity (S)
β-lactams	cefotaxime (CTX)	30	≤14	15-17	≥18
	cefaclor (CEC)	30	≤14	15-17	≥18
	meropenem (MEM)	10	≤28	29-37	≥38
	Cefradine (CE)	30	≤14	15-17	≥18
	ampicillin (AMP)	10	≤13	14-16	≥17
Aminoglycosides	Amikacin (AK)	30	≤14	15-16	≥17
	neomycin (N)	30	≤12	13-16	≥17
Quinolones	Ciprofloxacin (CIP)	5	≤15	16-22	≥23
	enrofloxacin (ENR)	10	≤21	22-28	≥29
Tetracyclines	tetracycline (TE)	30	≤14	15-18	≥19
	doxycycline (DOX)	30	≤12	13-15	≥16
Others	Compound sulfamethoxazole (SXT)	24	≤23	24-32	≥33
	rifampicin (RD)	5	≤16	17-19	≥20
	azithromycin (AZM)	15	≤13	14-17	≥18

	Florfenicol (FON)	30	≤12	13-17	≥18
	Polymyxin B (PB)	300	≤7	8-11	≥12

Table S4 Primers information of antibiotic resistance genes (ARGs)

Classification	Gene	Forward primer	Reverse primer
aminoglycoside	*aac*	CCCTGCGTTGTGGCTATGT	TTGGCCACGCCAATCC
aminoglycoside	*aac(3)- I*	ACCTACTCCCAACATCAGCC	ATATAGATCTCACTACGCGC
aminoglycoside	*aac(6')I1*	GACCGGATTAAGGCCGATG	CTTGCCTTGATATTCAGTTTTTATAACCA
aminoglycoside	*aac(6')-Ib(aka aacA4)-01*	GTTTGAGAGGCAAGGTACCGTAA	GAATGCCTGGCGTGTTTGA
aminoglycoside	*aac(6')-Ib(aka aacA4)-02*	CGTCGCCGAGCAACTTG	CGGTACCTTGCCTCTCAAACC
aminoglycoside	*aac(6')-Ib(aka aacA4)-03*	AGAAGCACGCCCGACACTT	GCTCTCCATTCAGCATTGCA
aminoglycoside	*aac(6')-II*	CGACCCGACTCCGAACAA	GCACGAATCCTGCCTTCTCA
aminoglycoside	*aac(6')-Iy*	GCTTTGCGGATGCCTCAAT	GGAGAACAAAAATACCTTCAAGGAAA
aminoglycoside	*ant（3"）-I*	TGATTTGCTGGTTACGGTGAC	CGCTATGTTCTCTTGCTTTTC
aminoglycoside	*aph*	TTTCAGCAAGTGGATCATGTTAAAAT	CCAAGCTGTTTCCACTGTTTTTC
aminoglycoside	*aph(2')-Id-01*	TGAGCAGTATCATAAGTTGAGTGAAAAG	GACAGAACAATCAATCTCTATGGAATG
aminoglycoside	*aph6ia*	CCCATCCCATGTGTAAGGAAA	GCCACCGCTTCTGCTGTAC
aminoglycoside	*aphA1(aka kanR)*	TGAACAAGTCTGGAAAGAAATGCA	CCTATTAATTTCCCCTCGTCAAAAA
aminoglycoside	*aphA3-01*	TGACGATCCGAGGAGTATC	AGCTGGTGAACGTACTAC
aminoglycoside	*aphA3-02*	CAATCGGATCGTCAAGCGATC	ATGCTACTTGAGCTTAGGCGAAA
fca	*acrA-03*	CAGACCCGCATCGCATATT	CGACAATTTCGCGCTCATG
integron	*intl-1*	CGAACGAGTGGCGGAGGGTG	TACCCGAGAGCTTGGCACCCA
integron	*Intl2-01*	GACGAATAATCGTGACGACGAT	ACTTTCTCGGTCTGCCATGAG
integron	*Intl2-02*	AACGTAGCTCTGAAAACGCAGTT	TATGTGACCTGGCGATGATTTGAC
integron	*Intl3-01*	CCGAAAAGAAGGGATTAAGCGAG	AACGGTTTACGGAACGTACGACC
integron	*Intl3-02*	ATCGGAAATCGATTCGGACT	ATGGCAATCGATCGAATTCA
MLSB	*ermA*	TTGAGAAGGGATTTGCGAAAAG	ATATCCATCTCCACCATTAATAGTAAACC
MLSB	*ermB*	TAAAGGGCATTTAACGACGAAACT	TTTATACCTCTGTTTGTTAGGGAATTGAA
MLSB	*ermC*	TTTGAAATCGGCTCAGGAAAA	ATGGTCTATTTCAATGGCAGTTACG
MLSB	*mcr1*	CGCGATGCTACTGATCACCA	GGTCGTATCATAGACCGTGCC
MLSB	*mcr2*	AATCGTACCCAAGTAATC	TAGGCTGAAACTGTCCTAAATCG
MLSB	*msrA-01*	CTGCTAACACAAGTACGATTCCAAAT	TCAAGTAAAGTTGTCTTACCTACACCATT
other	*IS26*	TACCGTAAGGCTACAACGT	AACTGAGCTAGCTCCTTTACGGC
other	*IS3*	TCCGGCTAGTCAATTCGGACCGA	TACGTAAAGGCTAGCTTACG
other	*IS613*	AGGTTCGGACTCAATGCAACA	TTCAGCACATACCGCCTTGAT
other	*ISCR1*	ACTGGCAAACGGTCATCG	ACTGTCCGATCCCTTACTGAAACT
other/efflux	*qacA*	TGGCAATAGGAGCTATGGTGTTT	AAGGTAACACTATTTTCGGTCCAAATC
other/efflux	*qacEdelta1-01*	TCGCAACATCCGCATTAAAA	ATGGATTTCAGAACCAGAGAAAGAAA
other/efflux	*qacEdelta1-02*	CCCCTTCCGCCGTTGT	CGACCAGACTGCATAAGCAACA
other/efflux	*qacH-01*	GTGGCAGCTATCGCTTGGAT	CCAACGAACGCCCACAA
other/efflux	*qacH-02*	GTGGCAGCTATCGCTTGGAT	TGAACGCCCAGAAGTCTAGTTTT
quinilone	*acrA-01*	CAACGATCGGACGGGTTTC	TGGCGATGCCACCGTACT
quinilone	*acrA-02*	GGTCTATCACCCTACGCGCTATC	GCGCGCACGAACATACC
quinilone	*acrB-01*	AGTCGGTGTTCGCCGTTAAC	CAAGGAAACGAACGCAATACC
quinilone	*qnrA*	AGGATTTCTCACGCCAGGATT	CCGCTTTCAATGAAACTGCAA
tetracycline	*tcrB*	GCAACTGCCTAGCTAGCTTAACG	GGCATGCTGGGTCAAGCCTTCAC
tetracycline	*tetA-01*	GCTGTTTGTTCTGCCGGAAA	GGTTAAGTTCCTTGAACGCAAACT
tetracycline	*tetA-02*	CTCACCAGCCTGACCTCGAT	CACGTTGTTATAGAAGCCGCATAG
tetracycline	*tetB-01*	AGTGCGCTTTGGATGCTGTA	AGCCCCAGTAGCTCCTGTGA
tetracycline	*tetB-02*	GCCCAGTGCTGTTGTTGTCAT	TGAAAGCAAACGGCCTAAATACA
vancomycin	*vanA*	AAAAGGCTCTGAAAACGCAGTTAT	CGGCCGTTATCTTGTAAAAACAT
vancomycin	*vanB-01*	TTGTCGGCGAAGTGGATCA	AGCCTTTTTCCGGCTCGTT
vancomycin	*vanC-01*	ACAGGGATTGGCTATGAACCAT	TGACTGGCGATGATTTGACTATG
β-lactamase	*blaCMY2-01*	AAAGCCTCAT GGGTGCATAAA	ATAGCTTTTGTTTGCCAGCATCA
β-lactamase	*blaCMY2-02*	GCGAGCAGCCTGAAGCA	CGGATGGGCTTGTCCTCTT
β-lactamase	*blaCTX-M-01*	GGAGGCGTGACGGCTTTT	TTCAGTGCGATCCAGACGAA
β-lactamase	*blaCTX-M-02*	GCCGCGGTGCTGAAGA	ATCGGATTATAGTTAACCAGGTCAGATTT
β-lactamase	*blaCTX-M-03*	CGATACCACCACGCCGTTA	GCATTGCCCAACGTCAGATT
β-lactamase	*blaCTX-M-04*	CTTGGCGTTGCGCTGAT	CGTTCATCGGCACGGTAGA
β-lactamase	*blaCTX-M-05*	GCGATAACGTGGCGATGAAT	GTCGAGACGGAACGTTTCGT
β-lactamase	*blaCTX-M-06*	CACAGTTGGTGACGTGGCTTAA	CTCCGCTGCCGGTTTTATC
β-lactamase	*blaGES*	GCAATGTGCTCAACGTTCAAG	GTGCCTGAGTCAATTCTTTCAAAG
β-lactamase	*blaIMP-01*	AACACGGTTTGGTGGTTCTTGTA	GCGCTCCACAAACCAATTG
β-lactamase	*blaKPC*	ATGTCACTGTATCGCCGTCTA	TTACTGCCCGTTGACGCCCAA
β-lactamase	*blaMOX/blaCMY*	CTATGTCAATGTGCCGAAGCA	GGCTTGTCCTCTTTCGAATAGC
β-lactamase	*BlaNDM*	CTTGAGGTTGCTTGCCG	AGCGTCAGAATTGCCCATACT

β-lactamase	*blaOCH*	GGCGACTTGCGCCGTAT	TTTTCTGCTCGGCCATGAG
β-lactamase	*blaOXA1/blaOXA30*	CGGATGGTTTGAAGGGTTTATTAT	TCTTGGCTTTTATGCTTGATGTTAA
β-lactamase	*blaOXA10-01*	CGCAATTATCGGCCTAGAAACT	TTGGCTTTCCGTCCCATTT
β-lactamase	*blaOXA10-02*	CGCAATTATCGGCCTAGAAACT	TTGGCTTTCCGTCCCATTT
β-lactamase	*blaOXY*	CGTTCAGGCGGCAGGTT	GCCGCGATATAAGATTTGAGAATT
β-lactamase	*blaPER*	TGCTGGTTGCTGTTTTTGTGA	CCTGCGCAATGATAGCTTCAT
β-lactamase	*blaSHV-01*	TCCCATGATGAGCACCTTTAAA	TTCGTCACCGGCATCCA
β-lactamase	*blaSHV-02*	CTTTCCCATGATGAGCACCTTT	TCCTGCTGGCGATAGTGGAT
β-lactamase	*blaTEM*	AGCATCTTACGGATGGCATGA	TCCTCCGATCGTTGTCAGAAGT
β-lactamase	*blaVEB*	CCCGATGCAAAGCGTTATG	GAAAGATTCCCTTTATCTATCTCAGACAA
β-lactamase	*blaVIM*	GCACTTCTCGCGGAGATTG	CGACGGTGATGCGTACGTT
